# Survey of the approach to the diagnosis and management of bacterial pneumonia in adult horses by equine veterinarians

**DOI:** 10.3389/fvets.2024.1484970

**Published:** 2024-12-23

**Authors:** Kate L. Hepworth-Warren, Kim Love

**Affiliations:** ^1^Department of Clinical Sciences, College of Veterinary Medicine, North Carolina State University, Raleigh, NC, United States; ^2^Consultant, Athens, GA, United States

**Keywords:** pleuropneumonia, pneumonia, antimicrobial stewardship, equine respiratory disease, survey

## Abstract

Bacterial pneumonia is a common disease in adult horses, but there are no guidelines for practitioners regarding risk factors, diagnosis, and management of the disease. The objectives of this study were to describe how a group of equine veterinarians diagnose and treat bacterial pneumonia in adult horses. A 22-question survey was distributed via multiple platforms to equine veterinarians asking questions regarding the frequency with which they identified specific clinical findings and used certain diagnostic modalities, and the approach to antimicrobial and adjunct therapy. Three hundred nine survey responses were received of which 244 were complete and used in the final analyses. There are significant differences in the ways that different equine veterinarians diagnose and treat bacterial pneumonia based on practice type and training of the practitioner. The majority of practitioners treat with antimicrobials for longer than 2 weeks, and only 53.3% of respondents reported that they “always” or “most of the time” used culture and antimicrobial susceptibility testing to guide therapy. There is a need for guidelines to reduce the duration of therapy and improve antimicrobial stewardship when treating bacterial pneumonia in horses.

## Introduction

Bacterial pneumonia is common in horses and is diagnosed and treated by equine practitioners in general and specialty practice. Pneumonia develops as a sequela to long-distance transportation, aspiration, and viral upper respiratory disease and can be fatal if not diagnosed and treated promptly (1–5). While there are guidelines in human and small animal medicine for the diagnosis and management of pneumonia, no such guidelines exist in equine practice (6, 7).

Much of the current body of literature regarding bacterial pneumonia in adult horses is outdated, and little information is available describing how veterinarians treat bacterial pneumonia—thus new studies are needed to develop guidelines to standardize the management of bacterial pneumonia among equine veterinarians.

By developing guidelines for the management of bacterial pneumonia in horses, antimicrobial stewardship could be improved which benefits human and animal health. In ambulatory equine practice, respiratory disease is the third most common body system (8) for which antimicrobials are prescribed, making inappropriate antimicrobial use in pneumonia a potentially significant factor in the development of antimicrobial resistance. Factors that may play a role in poor antimicrobial stewardship in equine pneumonia include lack of culture and susceptibility testing, inappropriate use of protected classes of antimicrobials, and prolonged durations of therapy. Additionally, practitioners may lack a thorough understanding of pharmacokinetics, pharmacodynamics, and the spectrum of activity of different antimicrobials. In human medicine, and more recently small animal medicine, standard recommendations and guidelines have been created for therapeutic protocols which describe a shorter course of antimicrobial therapy than is typically reported in equine medicine (4, 6, 7). Multiple studies in these species have shown no benefit to extending antimicrobial therapy past these recommended times (9–11). While the effect of an abbreviated course of antimicrobial therapy on outcome in equine pneumonia has not been investigated, it is possible that more clear guidelines could be developed in the future to improve antimicrobial stewardship.

The objectives of this study were to describe clinical characteristics of pneumonia identified by equine practitioners, and the methods and therapies used to diagnose and treat the disease. We aimed to identify differences in these practices and their association with years of experience, specialty training, practice type, and geographic location of the practitioner and to identify patterns in antimicrobial selection. We hypothesized that there would be significant differences in the diagnostic techniques, the therapy prescribed, and the tools used to monitor clinical progression of pneumonia by practitioners with different levels of experience, training, practice types, and geographic location.

## Materials and methods

A 22-question survey (Supplementary material S1) was created in Qualtrics XM (Qualtrics, 2023, Qualtrics International Inc.) by the author and reviewed by multiple colleagues prior to disseminating the survey. All questions were formatted such that participants were required to answer each question before moving to the next and could not skip questions. Prior to distribution, the survey was approved by the NC State University Institutional Review Board (IRB #26273). Links to the survey were distributed to the American College of Veterinary Internal Medicine listserv to diplomates in Large Animal Internal Medicine, via the AAEP listserv to equine practitioners, and on Facebook. Links were made public and posted in Facebook groups open only to equine veterinarians but were also shared on practitioner’s personal pages. Colleagues of the author were asked to disseminate the link to other equine veterinarians. To the complete the survey, participants were asked to confirm that they were over the age of 18, and a licensed veterinarian who was either currently in equine practice or had been in the past. The survey was available in a desktop compatible form and mobile-friendly format and remained open from 8/9/23 to 10/24/23, after which time results were exported and statistical analyses performed.

### Statistical analysis

Summary statistics were prepared using IBM SPSS Statistics v. 29. Statistical analyses were performed using R software (version 4.3.2) using the FSA and tidyR packages (12, 13). Statistical significance was set at *p* < 0.05. For comparing rankings of responses to certain items (e.g., “which variable do you use to guide the duration of antimicrobial therapy?,” “how often do you use a specific treatment?”) Friedman’s test was used to determine whether there were overall differences. When these overall tests were statistically significant, the Nemenyi post-hoc test was used to determine which specific outcomes had significantly different rankings than others. For comparing responses to Likert-type items (with ordered responses) between groups, Mann Whitney *U* tests or Kruskal–Wallis tests (depending on whether 2 or more groups were compared) were used. When significant differences were identified among more than 2 groups, *post hoc* Dunn’s tests with a Holm correction for multiple testing were performed. For comparing responses to items with dichotomous (yes/no type) responses between groups, Fisher exact tests were used. When required, *post hoc* pairwise Fisher exact tests with a false discovery rate (FDR) adjustment were used to determine which groups were significantly different from which other groups. Associations between two ordinal variables (e.g., length of time in practice and duration of antimicrobial therapy) were identified using Spearman’s correlation.

## Results

A total of 309 responses were obtained, of which 244 were complete. Only complete survey responses were included in data analysis.

### Practitioner demographics and practice types

**Figure 1:**
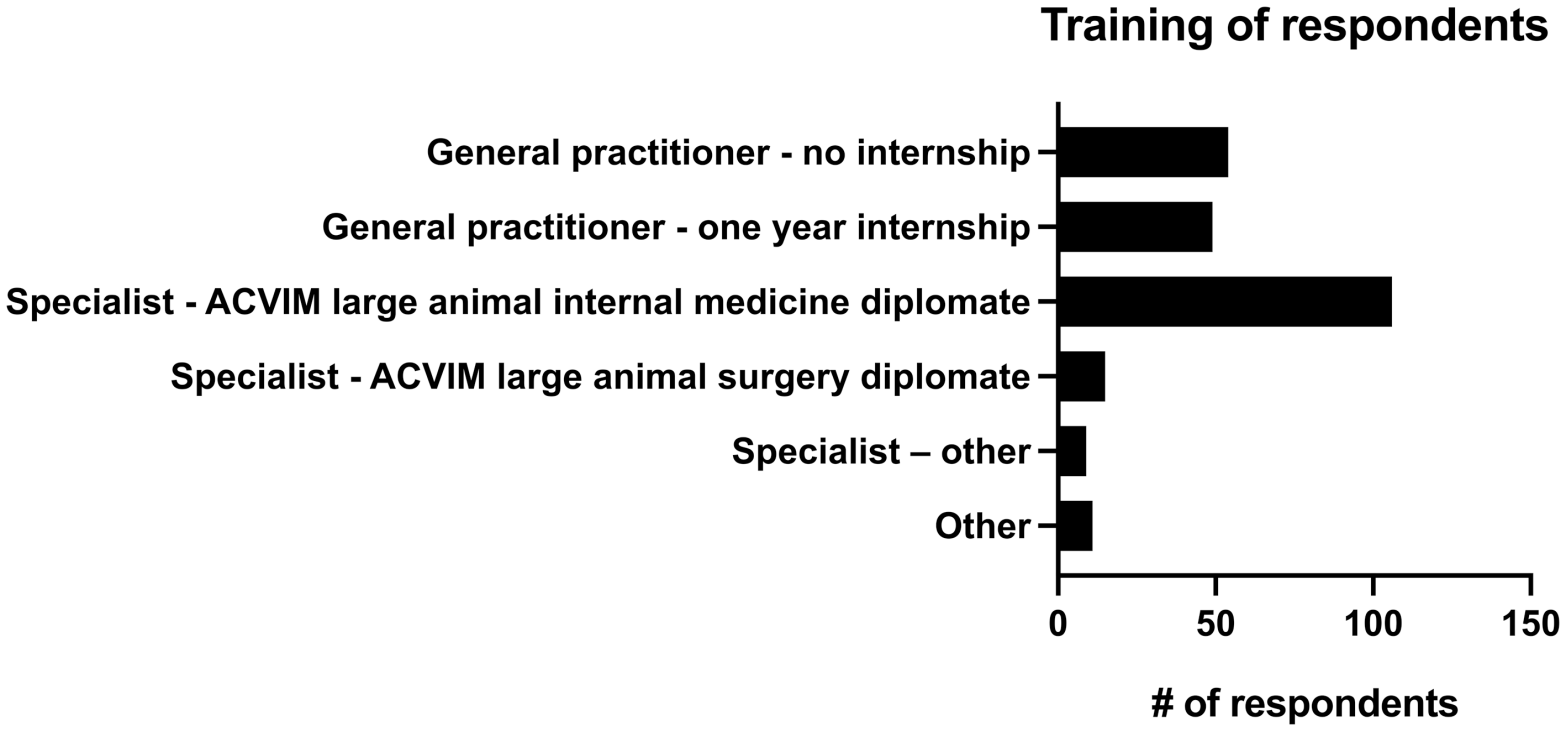
Level of training of 244 survey respondents.

**Figure 2:**
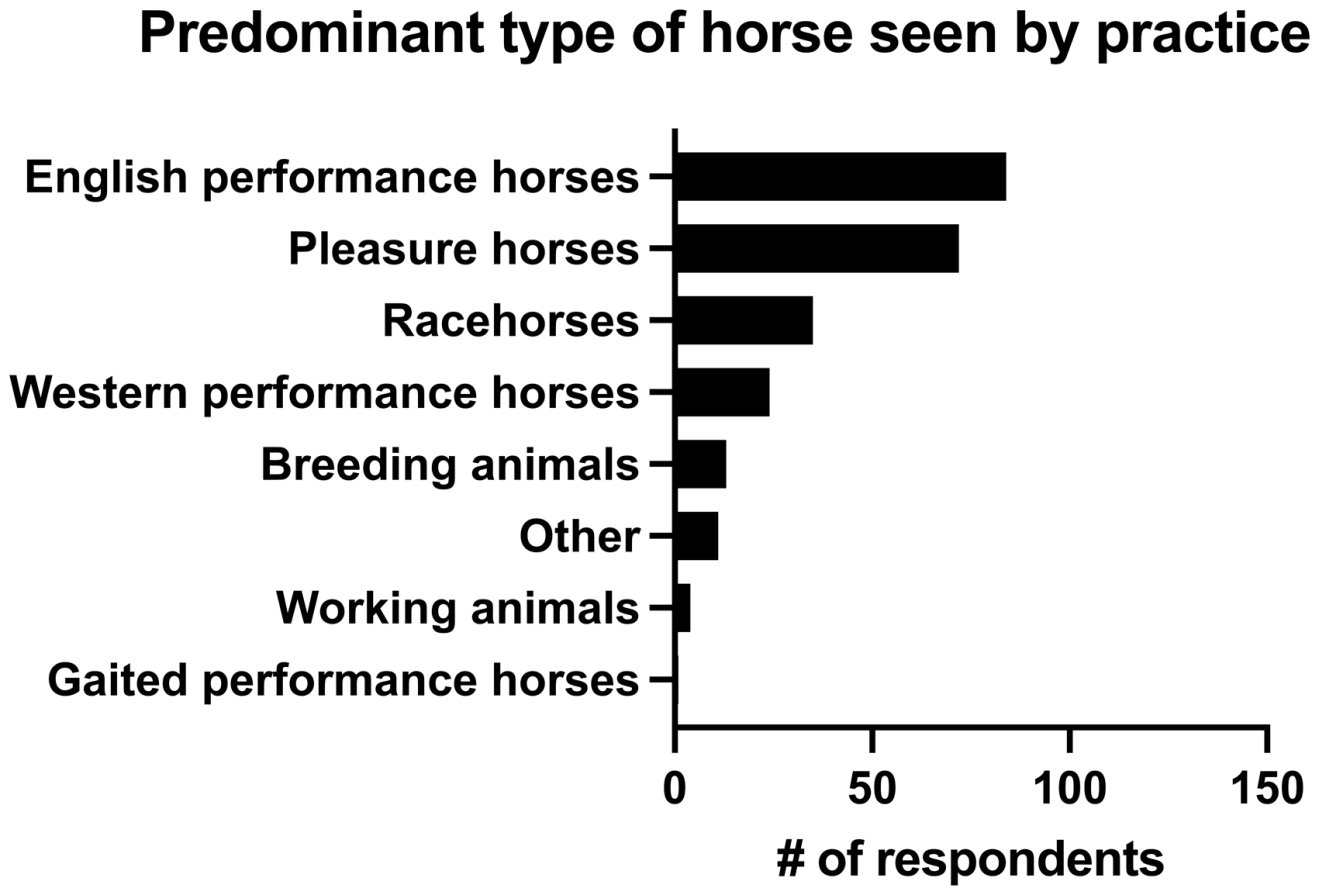
Predominant type of horse seen by the practice of 244 survey respondents.

13.9% of practitioners (*n* = 34/244) had 0–5 years of practice experience, 26.6% (*n* = 65/244) had 6–10 years of experience, 16.4% (*n* = 40/244) had 11–15 years of experience, 13.9% (*n* = 34/244) had 16–20 years of experience and 29.1% (*n* = 71/244) had greater than 20 years of experience. The majority of respondents (82.8%, *n* = 202/244) described their practice as being 76–100% equine, followed by those that saw 51–75% equine (12.7%, *n* = 31/244). 2.9% (*n* = 7/244) of respondents saw 26–50% equine, and 1.6% (*n* = 4/244) of respondents described their practice as 0–25% equine. There were 4 main practice types in which respondents worked. The most common was academic referral practice (28.3%, *n* = 69/244), followed by ambulatory with a hospital facility (27.0%, *n* = 66/244), ambulatory only (23.4%, *n* = 57/244), and private specialty or referral practice (21.3%, *n* = 52/244). Seven respondents selected “other” to describe their practice type. Overall, 46.7% (*n* = 114/244) of respondents were classified as non-specialists and 53.3% of respondents (*n* = 130/244) were classified as specialists (see Figure 1 for additional sub-classification). Respondents most commonly worked on English performance horses (34.4%, *n* = 84/244), pleasure horses (29.5%, *n* = 72/244) and racehorses (14.3%, *n* = 35/244). Complete results can be seen in Figure 2.

### Geographical location

**Figure 3:**
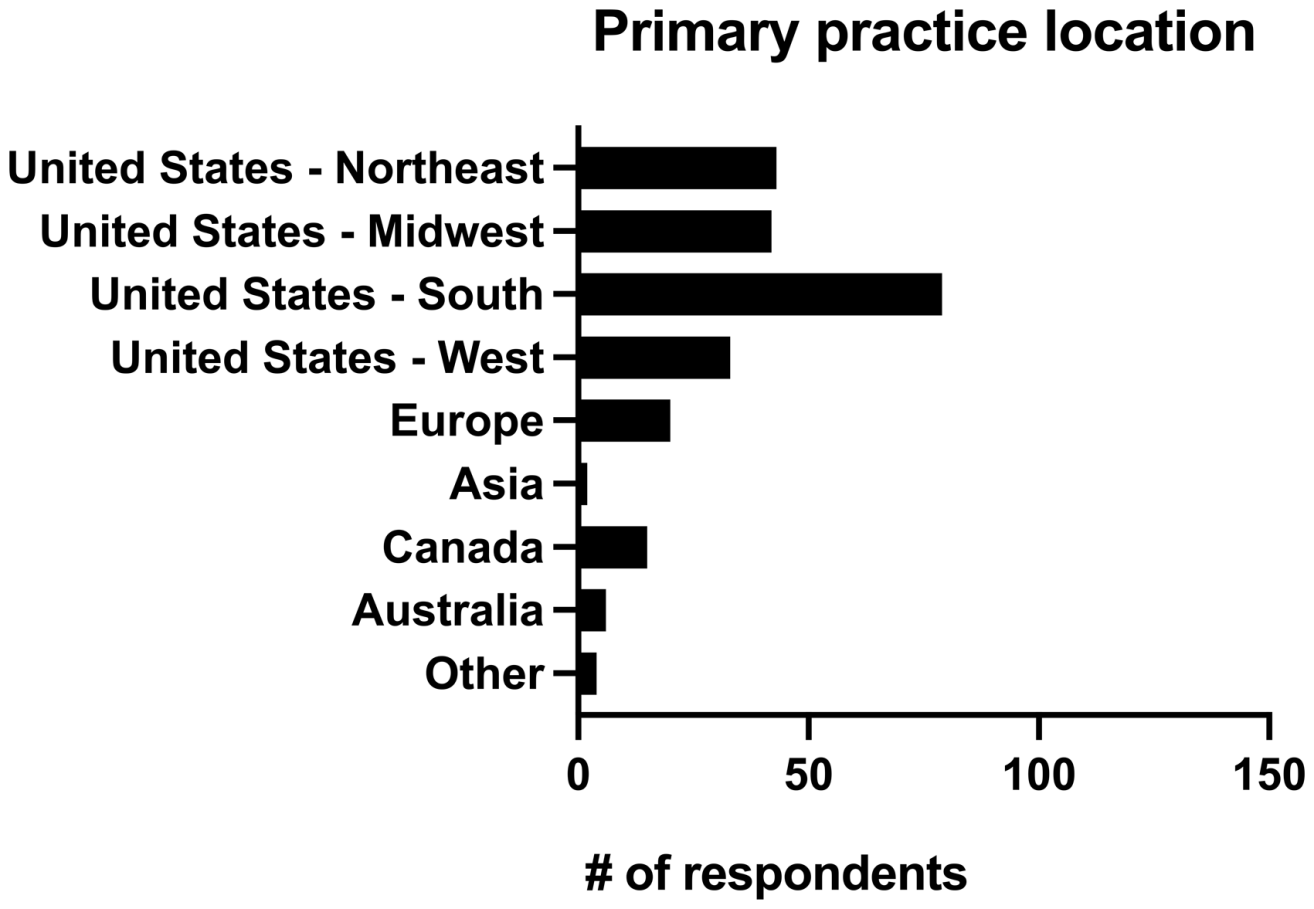
Primary practice location of 244 survey respondents.

Respondents were asked to identify their primary practice location and given the option of United States (Northeast, Midwest, South and West), Europe, Asia, Canada, Australia, and “other.” Practitioners in the United States made up 80.7% of respondents (*n* = 197/244). Four respondents (1.6%) worked in a primary location classified as other which included the Middle East (*n* = 2), the entire United States (*n* = 1), and Florida/California (*n* = 1). The geographic locations of all respondents can be seen in Figure 3.

### Clinical presentation and risk factors

**Figure 4:**
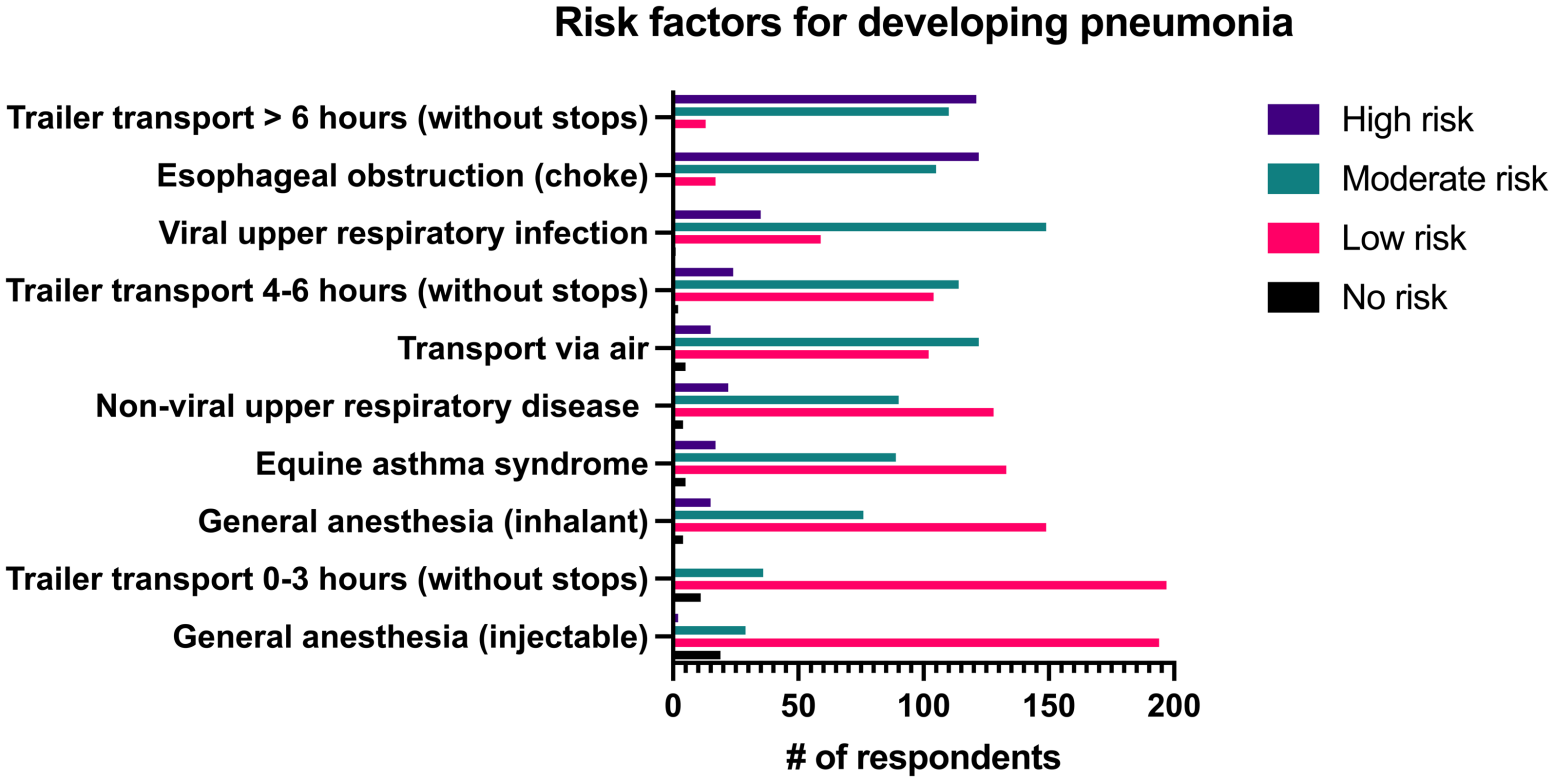
Reported level of risk associated with specific risk factors for development of pneumonia by 244 survey respondents.

Predisposing factors that were considered most frequently to place horses at high risk of developing pneumonia were trailer transport >6 h without stops (50%, *n* = 122/244) and esophageal obstruction (49.6%, *n* = 121/244). Items that were considered to place horses at moderate risk of developing pneumonia by the greatest number of respondents included viral upper respiratory infection (61.1%, *n* = 149/244), transportation via air (50%, *n* = 122/244), and trailer transport for 4–6 h without stopping (46.7%, *n* = 114/244). Trailer transportation for 0–3 h without stops (80.7%, *n* = 197/244), general anesthesia with injectable drugs (79.5%, *n* = 194/244), general anesthesia with inhalant (61.1%, *n* = 149/244), equine asthma syndrome (54.5%, *n* = 133/244), and non-viral upper respiratory diseases such as strangles, guttural pouch disease or laryngeal paralysis (52.5%, *n* = 128/244) were considered to be low risk by the majority of respondents. All risk factors can be seen in Figure 4.

**Figure 5:**
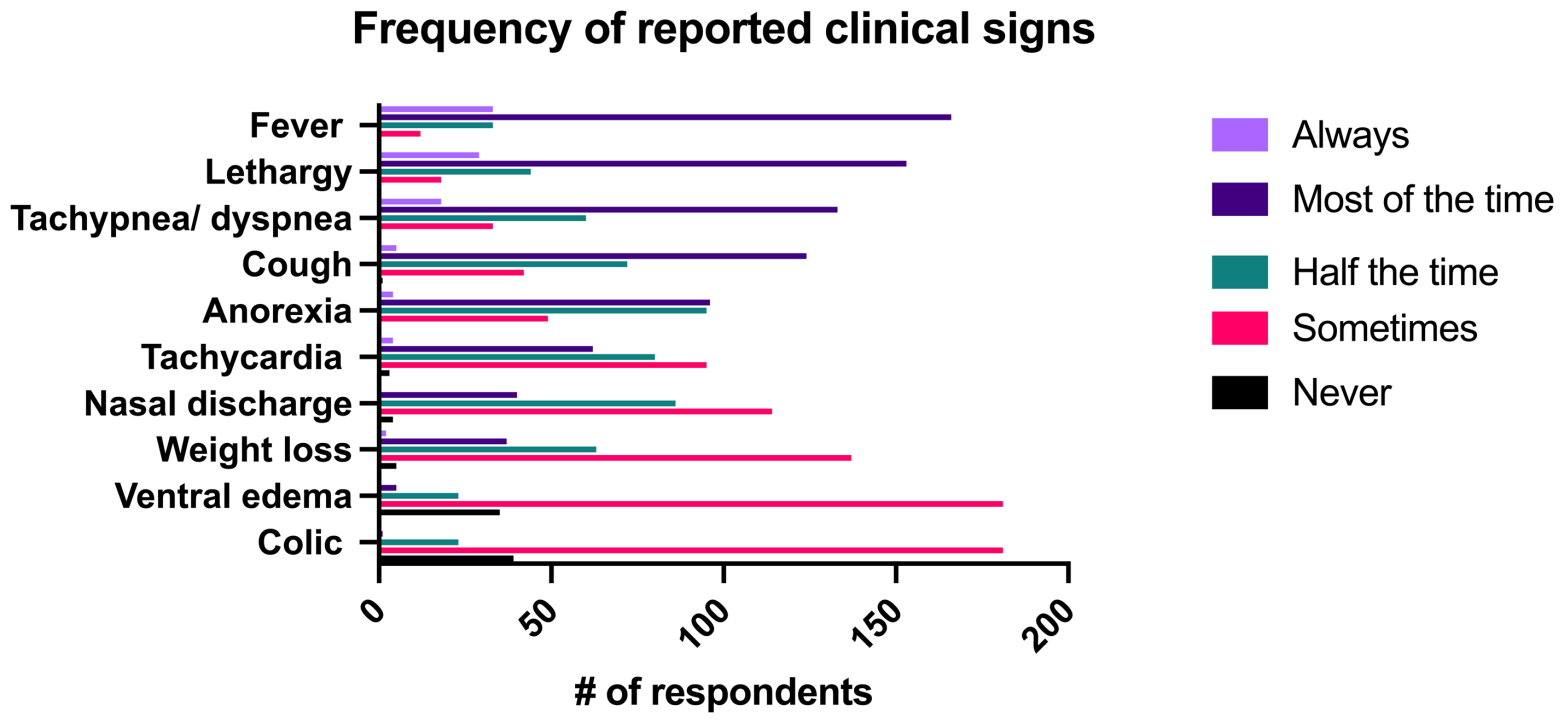
Reported frequency of clinical signs associated with pneumonia identified by 244 survey respondents.

Respondents were asked to describe how frequently certain clinical signs occurred with pneumonia. In the overall group, fever was described as being present “always” or “most of the time” in 81.5% (*n* = 199/244) of respondents, followed by lethargy (74.6% “always” or “most of the time,” *n* = 182/244) and tachypnea/dyspnea/increased respiratory effort (61.9% “always” or “most of the time,” *n* = 151/244). Fever (rectal temperature >101.5°F) was reported to occur with significantly greater frequency than all other signs aside from lethargy. Lethargy was reported to occur with significantly greater frequency than all remaining signs aside from fever and tachypnea/dyspnea. All clinical signs can be seen in Figure 5.

**Figure 6:**
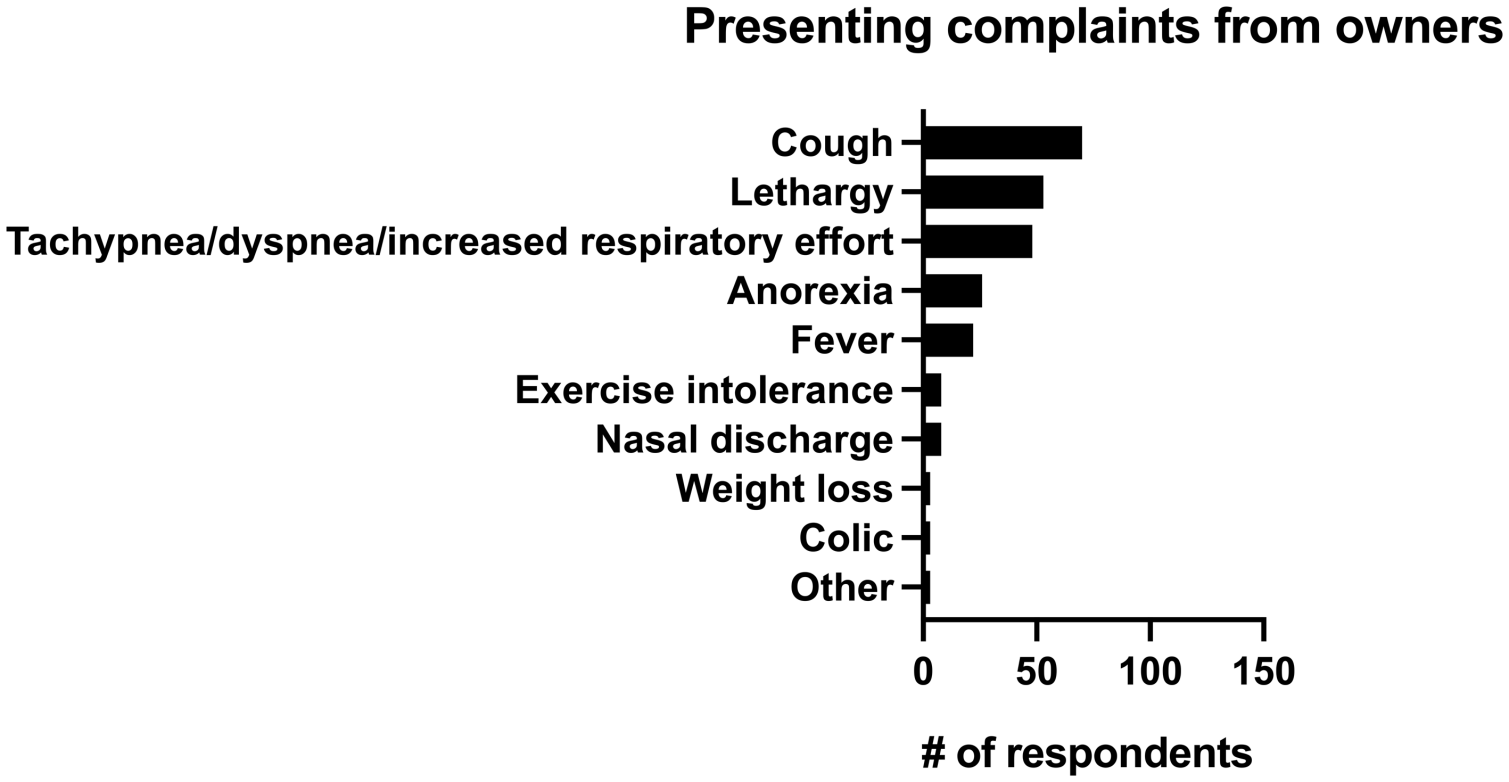
Primary reported presenting complaints from owners for horses with pneumonia identified by 244 survey respondents.

Respondents were also asked what the single most common presenting complaint with pneumonia was. The most common presenting complaint to respondents from clients was cough (28.7%, *n* = 70/244), followed by lethargy (21.7%, *n* = 53/244), and increased respiratory rate or effort (19.7%, *n* = 48/244). All presenting complaints can be seen in Figure 6.

### Diagnostics

**Figure 7:**
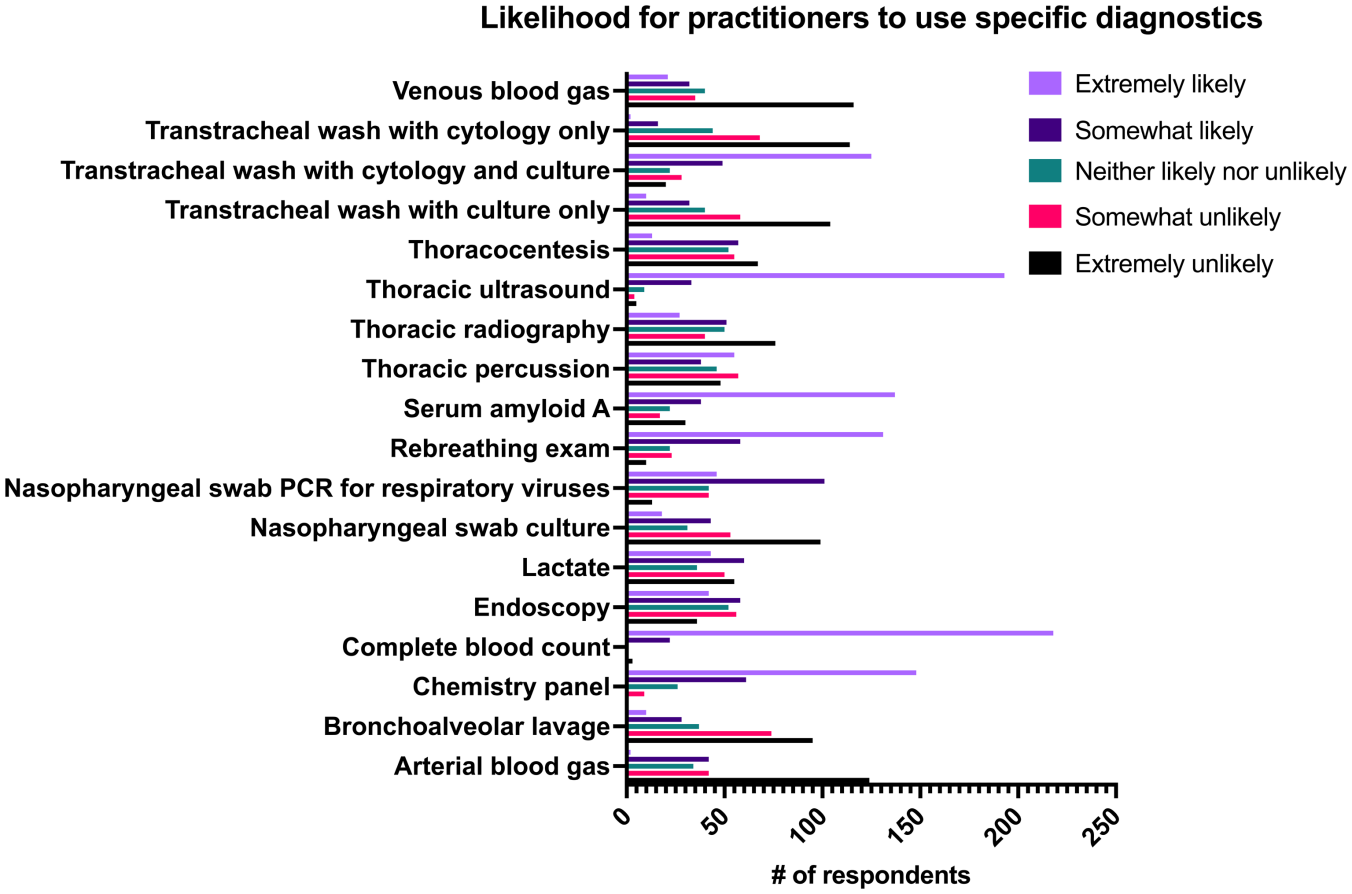
Likelihood of 244 practitioners to use individual diagnostics in cases of pneumonia in adult horses.

Respondents were asked how frequently they performed the following diagnostics when they suspected pneumonia: thoracic ultrasonography (US), thoracic radiography, endoscopy, CBC, chemistry panel, serum amyloid A (SAA), venous blood gas, arterial blood gas, L-lactate, tracheal wash with culture and cytology, tracheal wash with cytology only, bronchoalveolar lavage (BAL), thoracocentesis bacterial culture of nasopharyngeal swab, viral PCR of nasopharyngeal swab, rebreathing examination and thoracic percussion. Results can be seen in Figure 7. When respondents were questioned on the clinical findings that they used to classify pneumonia, exam findings (98.8%, *n* = 241/244), thoracic ultrasonography (94.3%, *n* = 230/244), and presence of inflammatory leukogram or hyperfibrinogenemia on CBC (91.4%, *n* = 223/244) were the most commonly used. The presence of bacterial grown on culture of tracheal wash was used to classify pneumonia by 68.9% (*n* = 151/244) of respondents and the presence of suppurative inflammation on cytology of tracheal wash was used by 61.9% (*n* = 151/244) of respondents.

Respondents were additionally asked about their comfort performing four procedures. The majority of respondents reported that they were very comfortable performing thoracic ultrasound (US) (76.6%, *n* = 187/244), tracheal wash (67.6%, *n* = 165/244), bronchoalveolar lavage (63.1%, *n* = 154/244), and thoracocentesis (54.1%, *n* = 132/244). 18.9% of respondents reported that they were extremely uncomfortable (18.9%, *n* = 46/244) performing thoracocentesis.

Tracheal washes were performed significantly more frequently at referral practices (private practice and academic) than primarily ambulatory or combined ambulatory and hospital practices. There was no significant difference between use of tracheal washes between academic and private referral practices. Specialists were significantly more likely (*p* < 0.001) than non-specialists to perform tracheal washes. There were no significant differences in likelihood of performing tracheal washes between practices when categorized by primary type of horse seen. Tracheal washes were significantly more likely to be performed by respondents in Europe than Canada and all regions of the United States. The majority of respondents performed tracheal washes percutaneously as their primary method (50%, *n* = 122/244). Endoscopic tracheal washes were the main technique in 35.2% of respondents (*n* = 86/244), whereas 14.8% of respondents (*n* = 36/244) reported they did not typically perform tracheal washes. The initial version of the survey only had 2 response options for the question of “If you perform tracheal washes, which method to you use most frequently?”—which was transcutaneous tracheal wash or endoscopic tracheal wash. A third option was added after shortly after the survey went live: “I do not perform tracheal washes” to avoid false reporting. Eighteen responses were submitted before the third option was available.

When the likelihood to use individual diagnostics was correlated with the duration of time respondents had been in practice, thoracic US (*p* = 0.019) and venous blood gas (*p* = 0.013) were significantly less likely to be used by practitioners that had been practicing for longer periods of time, whereas the likelihood for endoscopy to be used increased significantly (*p* = 0.004) with increased duration of practice experience.

Practitioners in academic or private referral hospitals were significantly more likely to use thoracic radiographs, tracheal washes, L-lactate (all *p*-values <0.001) and blood gases (venous and arterial, all *p*-values ≤0.01) than other practice types whereas respondents in all practice types (ambulatory and private referral hospitals) were significantly more likely to use SAA than practitioners in academic hospitals (all p values ≤0.006). Respondents in ambulatory practice were more likely to use rebreathing exams than referral type practices (*p* < 0.004). There were no significant differences between respondents in different practice settings and their likelihood of using thoracic US as a diagnostic. Respondents in private referral practices were significantly more likely to use endoscopy than practitioners in total (*p* = 0.016) or partial ambulatory practices (*p* = 0.016). There was a significantly greater proportion of respondents in partial ambulatory practices than in academic practice who reported using bronchoalveolar lavages as a diagnostic (*p* = 0.03). Practitioners in either private referral practice or academic practice were significantly more likely (*p* < 0.001) than practitioners in ambulatory practice to perform thoracic radiographs. Any practitioner with access to a hospital facility (academic, private referral or partial ambulatory) was significantly more likely to perform thoracocentesis or tracheal wash than an ambulatory practitioner (all *p*-values ≤0.003). Practitioners that work in ambulatory settings (total or partial) were significantly more likely to use nasopharyngeal swab cultures than practitioners in referral hospital settings (all *p*-values ≤0.026) and practitioners in total ambulatory practices were significantly more likely to use nasopharyngeal swabs with PCR testing for viruses than practitioners in academic settings (*p* < 0.022).

When diagnostic use was compared between specialists and non-specialists, non-specialists were significantly more likely to include SAA (*p* = 0.045), rebreathing examination (*p* = 0.013), bronchoalveolar lavage (*p* = 0.048) and nasopharyngeal swab cultures (*p* < 0.001) in the diagnostic work up for pneumonia whereas specialists were significantly more likely to utilize L-lactate (*p* < 0.001), arterial or venous blood gas (*p* < 0.001), thoracic US (*p* < 0.001) radiography (*p* < 0.001), endoscopy (*p* < 0.001), thoracocentesis (*p* < 0.001), and tracheal wash with cytology and culture (*p* < 0.001). When diagnostic use was evaluated by the primary type of horse seen by the respondent, endoscopy was significantly more likely to be used in racehorse dominant practices when compared to all other categories except for breeding animals (all *p*-values ≤0.004). When compared by geographic regions, practitioners in Europe were significantly more likely than practitioners in all four regions of the USA to use endoscopy in the diagnosis of pneumonia (all *p*-values ≤0.004).

### Antimicrobial therapy

#### Class of antimicrobial prescribed

**Figure 8:**
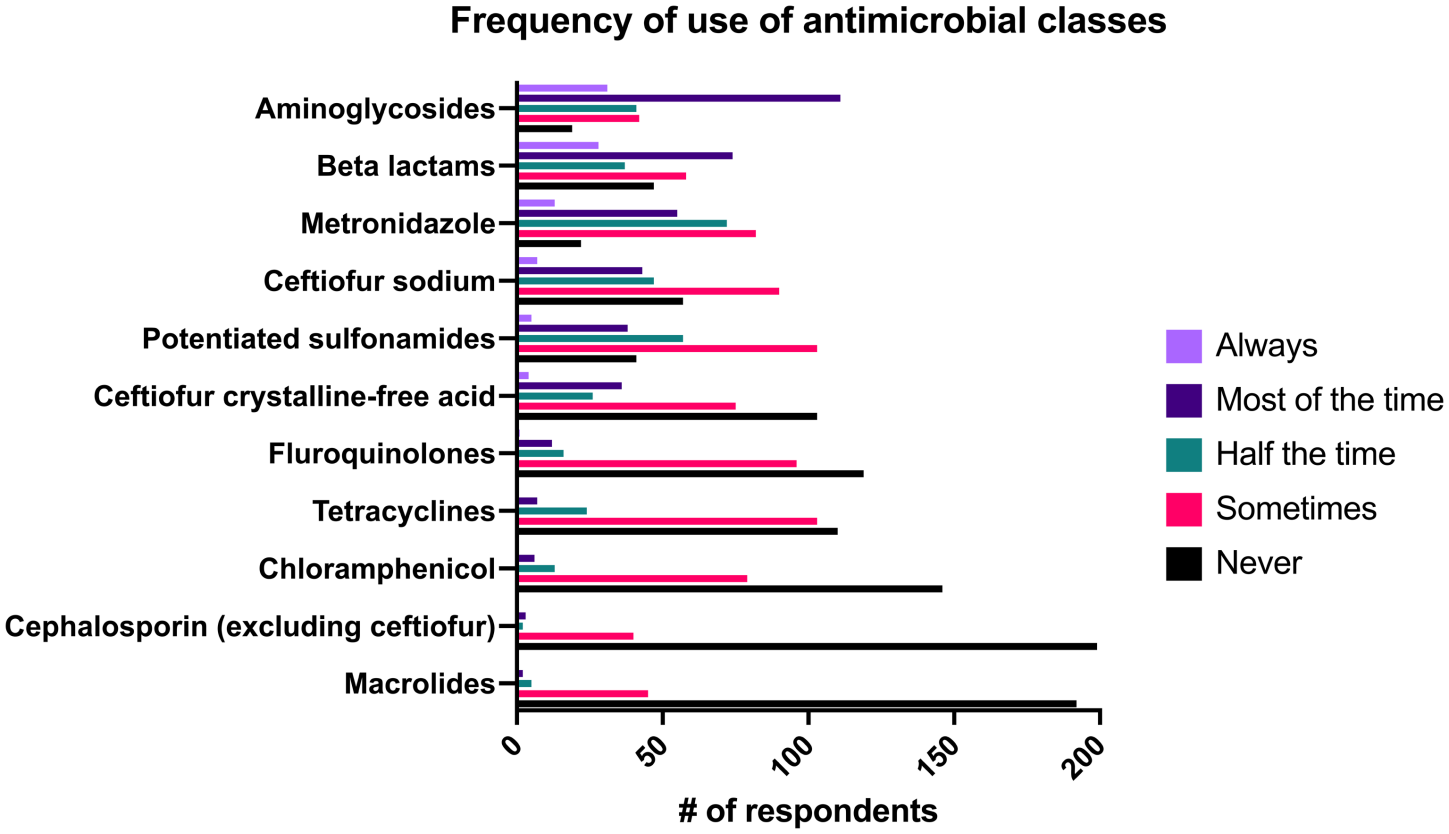
Reported frequency with which 244 survey respondents use specific classes of antimicrobials when treating bacterial pneumonia in adult horses.

The likelihood that practitioners would prescribe drugs in individual antimicrobial classes can be seen in Figure 8. Aminoglycosides were the most commonly prescribed, with 58.2% (*n* = 142/244) of respondents “always” or “most of the time” prescribing this class, followed by beta lactams (41.8%, *n* = 102/244) and metronidazole (27.8%, *n* = 68/244). There was a significant association of the length of time in practice and the use of potentiated sulfonamides (*p* = 0.022), macrolides (*p* = 0.013), chloramphenicol (*p* = 0.008), and carbapenems (*p* = 0.001). All of these classes had a significant positive correlation between their use and the duration of time the respondent had been in practice except for potentiated sulfonamides which was negatively correlated with length of time in practice. Additionally, respondents in ambulatory private practice were significantly more likely to prescribe potentiated sulfonamides than respondents in hospital or referral settings (*p* = 0.001). Similarly, respondents in ambulatory practices were significantly more likely to prescribe ceftiofur crystalline free acid (CFA) when compared to respondents in all types of referral hospitals (*p* < 0.001) and less likely to prescribe all beta lactams (*p* < 0.001) and aminoglycosides (*p* < 0.001) than those in referral practices. Respondents in all ambulatory practice were significantly less likely to prescribe metronidazole than respondents in academic practice (*p* < 0.001) and private referral centers (*p* = 0.025). Similarly, respondents in ambulatory practice with a clinic were significantly less likely than those in academic practice (p < 0.001) and private referral practice (*p* = 0.021) to prescribe metronidazole.

When comparing prescribed classes of antimicrobials between the primary type of horse seen in the practice, there were significant differences between the classes of antimicrobials prescribed by clinicians who worked mainly with racehorses compared to those who worked mainly with pleasure horses. Ceftiofur CFA (*p* = 0.02) and chloramphenicol (*p* = 0.04) were significantly more likely to be prescribed in primarily pleasure horse practices whereas aminoglycosides were significantly more likely to be prescribed in racehorse practices as compared to pleasure horse practices (*p* = 0.03).

#### Geographic regions

Veterinarians in all four regions of the USA were significantly more likely to prescribe ceftiofur CFA than veterinarians in Europe whereas veterinarians in Europe were significantly more likely to prescribe beta lactams than veterinarians in all regions of the USA except for the Midwest. European veterinarians were significantly more likely to prescribe aminoglycosides than veterinarians in the southern USA (*p* = 0.047), but there were no other differences associated with geographic area. Veterinarians in Canada were significantly more likely to prescribe tetracyclines than veterinarians in Europe (*p* = 0.001) and the southern USA (*p* = 0.014).

#### Culture and susceptibility

Culture and susceptibility results were always used by 9.4% (*n* = 23/244) of respondents when making final antimicrobial choices. 43.9% of respondents (*n* = 107/244) reported that they used these results “most of the time,” and 16% (*n* = 39/244) used the results “about half the time.” 25% (*n* = 61/244) of respondents “sometimes” used culture and susceptibility results and 5.7% (*n* = 14/244) reported that they “never” used this information in final antimicrobial selection. Veterinarians in academic (*p* ≤ 0.002) or private practice referral (*p* ≤ 0.004) settings were significantly more likely to base antimicrobial selection on culture and susceptibility testing than ambulatory-only practices as well as combination ambulatory/hospital facilities. Specialists are significantly more likely to base antimicrobial selection on culture results than non-specialists (*p* < 0.001). There were no significant differences between the how likely veterinarians were to base antimicrobial therapy on culture and susceptibility testing and the primary type of horse treated or the geographic region.

#### Duration of antimicrobial therapy

The most common duration of antimicrobial therapy was >2 weeks–4 weeks (50.4%, *n* = 123/244), followed by 1–2 weeks (38.5%, *n* = 94/244) and >4–8 weeks (9.4%, *n* = 23/244).

There was no statistically significant association between the duration of therapy and the number of years the respondent had been in practice (Spearman coefficient = −0.027, *p* = 0.68), nor was there a statistically significant difference in duration of prescribed therapy between different practice types (*p* = 0.131), primary type of horse seen in practice (*p* = 0.10), or geographic region (*p* = 0.26). Specialists were significantly more likely to treat for a longer length of time than non-specialists (*p* < 0.001).

In the overall group, normalization of total white blood cell (WBC) or neutrophil count and resolution of fever were used as the main criteria to guide duration of antimicrobial therapy by significantly more respondents than other variables. Resolution of ultrasonographic changes of the lungs was used as the next most frequent criterion by significantly more respondents than normalization of SAA or fibrinogen, resolution of cough, resolution of radiographic changes, or absence of bacterial growth on repeat tracheal aspirate and culture. The resolution of radiographic changes of the lungs and the absence of bacterial growth on repeat tracheal aspirate and culture were used by significantly fewer respondents than all other variables.

### Adjunct therapy in addition to systemic antimicrobials

**Figure 9:**
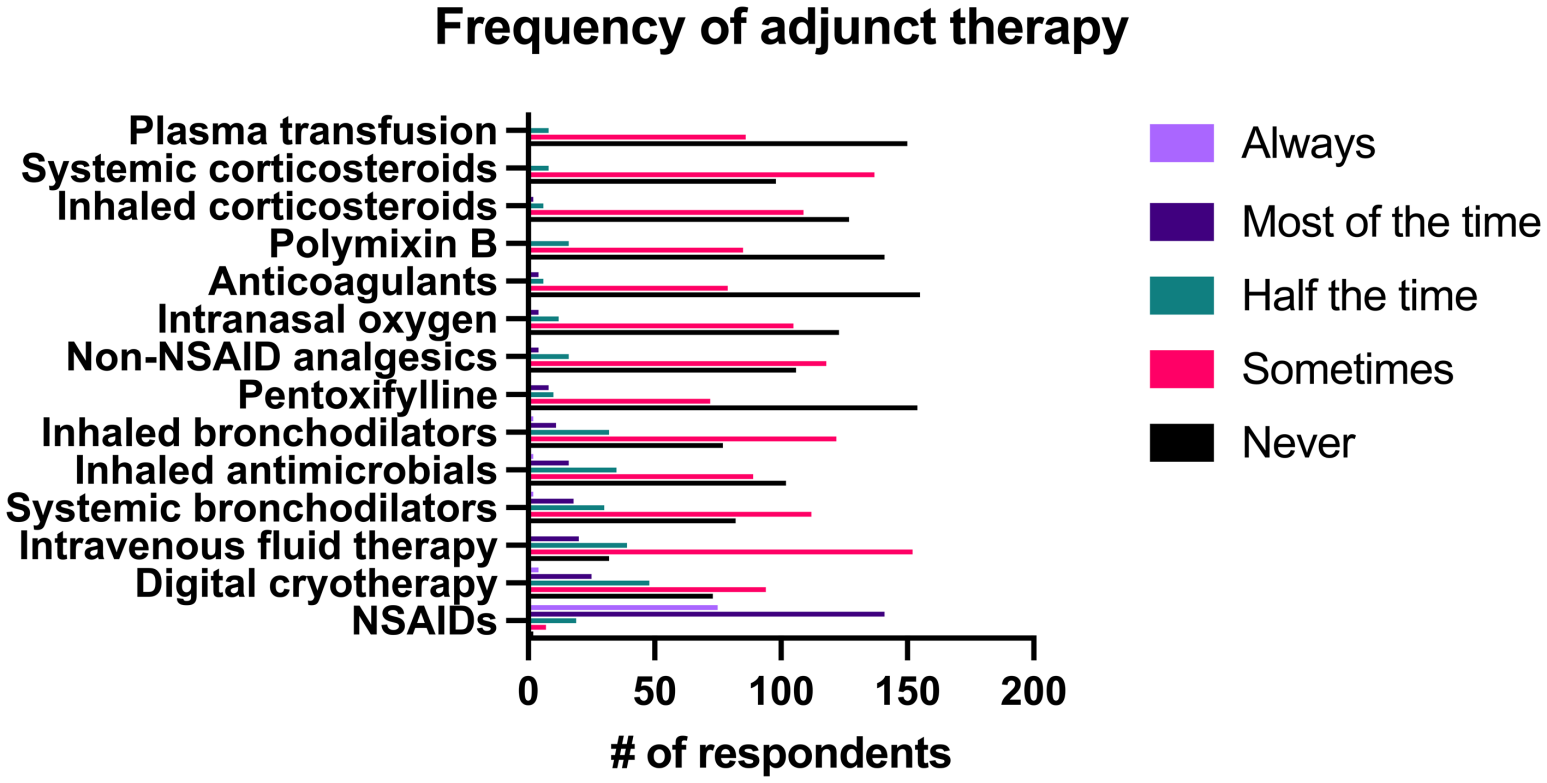
Reported frequency with which 244 survey respondents use different adjunct therapies when treating bacterial pneumonia in adult horses.

Out of all respondents, NSAIDs were prescribed significantly more frequently than all other adjunct therapies. Intravenous fluid therapy and digital cryotherapy were the next most common therapies and were used significantly more often than other therapies. Results of all adjunct therapy use can be seen in Figure 9.

When use of specific adjunct therapies was compared between specialists and non-specialists, there was no significant difference between groups in the frequency of use of NSAIDS (*p* = 0.56), systemic corticosteroids (*p* = 0.50), and inhaled corticosteroids (*p* = 0.61). Specialists were significantly more likely to administer intravenous fluid therapy, digital cryotherapy, inhaled bronchodilators, inhaled antimicrobials, non-NSAID analgesics, intranasal oxygen, polymyxin B, pentoxifylline, plasma transfusion, and anticoagulants (*p* < 0.001). Non-specialists were significantly more likely to prescribe inhaled bronchodilators than specialists (*p* = 0.002).

#### Treatment of pleuropneumonia

**Figure 10:**
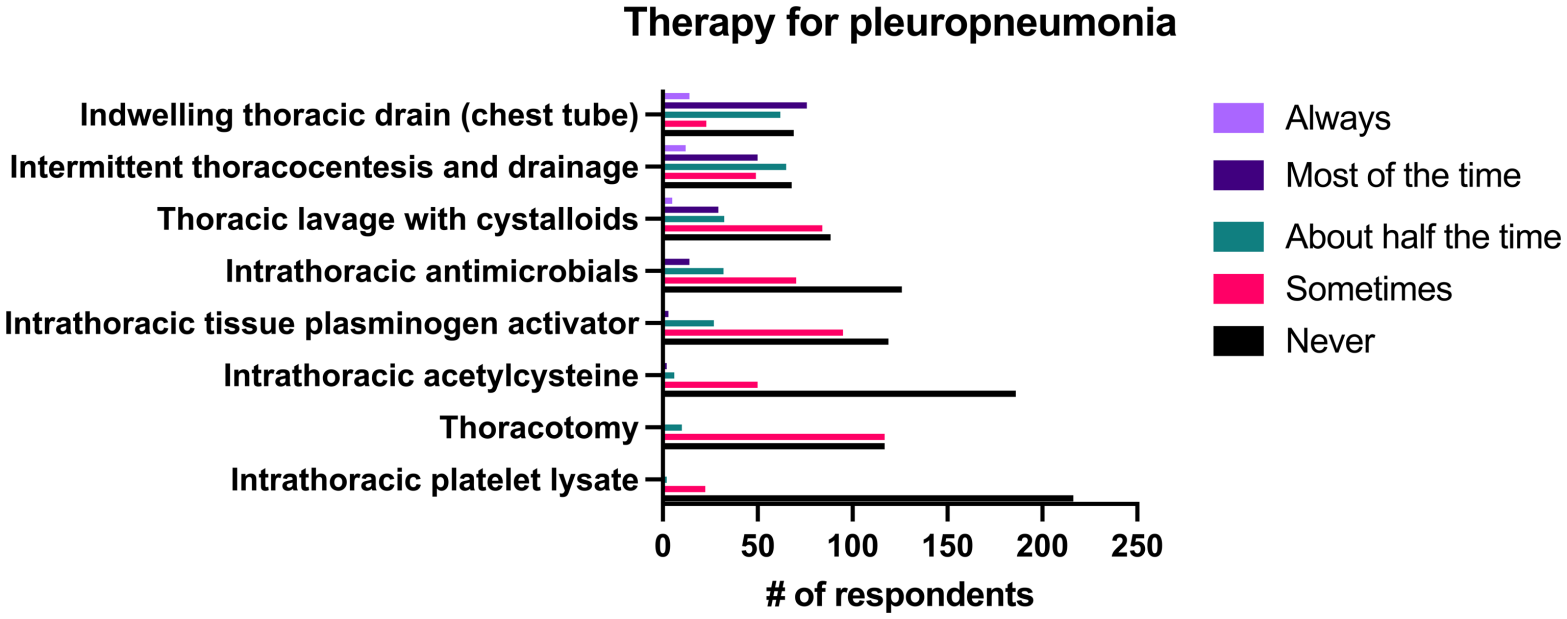
Reported frequency with which 244 survey respondents use specific therapies when treating pleuropneumonia in horses.

The most common treatment used in the management of pleuropneumonia was the placement of indwelling thoracic drains, which 36.8% (*n* = 90/244) of respondents reported they did “most of the time” or “always.” Intermittent thoracic lavage was used by 24.6% (*n* = 62/244) of respondents “most of the time” or “always. The majority of the therapies listed in the survey for management of pleuropneumonia were used “never” or “sometimes” by a large number of respondents. A complete list of responses can be seen in Figure 10.

## Discussion

Bacterial pneumonia is a common disease seen in equine practice, but its presentation, diagnosis and treatment can vary between practitioner training and experience, practice types, patient populations and geographic regions. The results of this survey highlight the differences between different groups of practitioners and illustrate the need for more clear guidelines in the identification and management of pneumonia to improve antimicrobial stewardship.

When clinicians are faced with vague and non-specific clinical signs, the patient’s history has a substantial effect on the differential diagnosis list. There are well-known risk factors that predispose adult horses to the development of pneumonia, including transportation, esophageal obstruction and to a lesser degree anesthesia and viral upper respiratory infection (1, 2, 4, 14, 15). Accordingly, 50% of respondents in this study reported that they considered trailer transportation >6 h and esophageal obstruction to be the historical factors that placed horses at highest risk for the development of pneumonia.

While travel is consistently noted as a risk factor in retrospective studies, the distance or duration of travel that increases the risk of development varies widely between studies, making it challenging for practitioners to known when to be concerned for an increased risk of pneumonia (1, 2, 14, 16–20). In 78 horses that developed pneumonia within 24 h of long-distance transportation, the horses were trailered 1,000–1,600 km, or 23–32 h (16). Another study described transportation >500 miles (>805 km) as a risk factor, whereas another compared travel duration of <8 h, 8–24 h, and > 24 h and found that horses that traveled for long periods of time (>24 h) and intermediate periods of time (8–24 h) were 113.9 and 15.7 times more likely, respectively, to develop respiratory disease during transportation than horses taken on short journeys (<8 h) (17, 19). Long distance transportation increases the risk for pneumonia and pleuropneumonia in horses because of the effect of stress on immune function and the effect of an elevated head position on the contamination of the lower airways and decreased mucociliary function (21, 22).

Esophageal obstruction is another major risk factor for the development of pneumonia as it results in aspiration of feed material and saliva and the development of secondary bacterial pneumonia in nearly half of affected horses (23, 24). In severe obstructions, intrathoracic rupture of the esophagus can occur leading to severe, usually fatal, pleuropneumonia (15). The degree of tracheal contamination is not consistently associated with the likelihood that pneumonia develops but horses that developed pneumonia following esophageal obstruction had a significantly longer duration of clinical signs of choke (median 18 h vs. 4 h) than those that did not (23, 24). In addition to esophageal obstruction and transportation, general anesthesia is often reported as a risk factor for pneumonia, however 62.7% (*n* = 153/244) of respondents considered it to present only a low risk or no risk. The frequency with which general anesthesia has been reported in the history of horses with pneumonia ranges from 0–23.9% (1–3, 14, 20, 25).

Fever (rectal temperature > 101.5° F) is often considered a hallmark of bacterial infections, yet it was only the fifth most common presenting complaint (9%, *n* = 22/244) described in this survey and was only classified as “always” being present by 13.5% (*n* = 33/244) of respondents though it was described as being present “most of the time” by 68% (*n* = 166/244) of respondents. It is important that pneumonia not be ruled out as a diagnosis solely based on the absence of fever as a recent retrospective identified fever in only 26% (*n* = 28/110) of confirmed cases of pneumonia and multiple studies have identified tachypnea and tachycardia as more common clinical signs (1, 14, 17, 26). While cough was the most frequently reported presenting complaint from clients (28.7%, *n* = 70/244), it was reported as being present “always” or “most of the time” by only 52.8% (*n* = 129/244) respondents. Similarly, nasal discharge was listed as the presenting complaint in 3.3% (*n* = 8/244) of respondents and was only seen as a clinical sign either “about half the time” or “sometimes” in 81.9% (*n* = 200/244) of respondents. Thus, it is critical that clinicians be aware that the absence of fever, nasal discharge, and cough not be used to rule out a diagnosis of pneumonia.

Many studies describing horses with bacterial pneumonia use positive bacterial growth on culture of tracheal fluid as a definitive diagnostic, but ultrasonography, culture of pleural fluid and BAL have also been reported (1, 2, 14, 26). The top three criteria used by the practitioner group as a whole in this study to confirm pneumonia were physical examination, thoracic ultrasonography and evidence of inflammation on CBC. From a list of 18 diagnostics, CBC was the most common diagnostic with 89.3% (*n* = 218/244) of respondents saying they were extremely likely to perform it. A large proportion of respondents also replied that they were extremely likely to use thoracic ultrasonography (79.1%, *n* = 193/244), chemistry panel (60.7%, *n* = 148/244) and serum amyloid A (56.1%, *n* = 131/244) when they suspected pneumonia. Only 15.6% of respondents reported that they were either “extremely likely” or “somewhat likely” to perform BAL when they suspected pneumonia. Bronchoalveolar lavage was used significantly more by respondents in ambulatory practices with clinic facilities than in academic hospitals (*p* = 0.028) and was also significantly more likely to be performed by non-specialists as compared to specialists (*p* = 0.048). These findings are likely due to clinical similarities between equine asthma syndrome and pneumonia, which often necessitates performance of multiple airway sampling techniques to reach the correct diagnosis. Bronchoalveolar lavage is considered the diagnostic of choice for equine asthma syndrome but is less appropriate as the primary diagnostic for pneumonia as it is typically performed by passing a non-sterile catheter through the nasopharynx leading to substantial contamination and thus inaccurate culture results. This is further complicated by the fact that horses with equine asthma syndrome may also have positive bacterial growth on a tracheal wash (27, 28). Additionally, cytology of BAL fluid (BALF) in horses with pneumonia is classified as normal >50% of the time despite positive cultures on tracheal wash (29, 30). Therefore, if pneumonia and equine asthma syndrome are both differential diagnoses, it is recommended that both BAL and tracheal washes performed, and that the tracheal sampling be performed first to maximize the yield of each diagnostic (27, 28).

Respondents were provided with a list of diagnostics and asked how likely they were to use each diagnostic modality when they suspected pneumonia (Figure 7). There were no significant differences between respondents in different practice settings and how likely they were to use CBC or thoracic ultrasonography, but there were significant differences between these groups when comparing the likelihood of using other diagnostics. L-lactate was measured more frequently by respondents in referral or hospital settings than by those in ambulatory practice (*p* < 0.001). Increased L-lactate at admission is associated with non-survival in horses with gastrointestinal disease and neonatal sepsis but there is a paucity of information available regarding any association between L-lactate and non-survival in horses with pneumonia thus its usefulness in these cases is unknown (1, 3, 14, 26, 31–33). Practitioners in private practice (all ambulatory, ambulatory with a clinic, private referral hospital) were significantly more likely to use SAA as a diagnostic tool than those in academic practice. Serum amyloid A is measured readily in the field using a stallside meter and serum concentration of SAA decreases significantly over the course of therapy in bacterial pneumonia (26). The major differences between practice settings and practitioner groups are likely attributable to the severity of cases seen in each setting, and differences in availability of equipment and technical support between hospital and ambulatory settings.

In ambulatory equine practice, respiratory disease is the third most common body system (24.4%) for which antimicrobials are prescribed (8). With increasing antimicrobial resistance, the judicial use of antimicrobial therapy in bacterial pneumonia is important to protect human and animal health. Ideally, antimicrobial selection should be based on culture and sensitivity, yet only 53.3% (*n* = 130/244) of respondents said that they either “always” or “most of the time” based antimicrobial choices on culture and susceptibility results. While the gold standard for antimicrobial therapy includes culture and susceptibility, and treatment for the shortest duration possible; ambulatory equine practice presents unique challenges that often makes this approach unrealistic (34). Despite 81.5% (*n* = 199/244) of respondents reporting that they were “somewhat comfortable” (13.9%) or “extremely comfortable” (67.6%) performing tracheal washes or aspirates, only 51.7% (*n* = 126/244) of respondents reported that they performed tracheal washes or aspirates “most of the time” or “always.” Veterinarians in the field may have little to no technical support, limited inventory in their vehicle, and clients may be more financially restricted than in a referral setting, leading to a lower likelihood for ambulator practitioners to perform these sampling procedures.

Though culture and susceptibility testing are the ideal way to make antimicrobial selections, clients may have financial limitations that preclude performance of a tracheal wash and thus empiric antimicrobial therapy is pursued without cytology or culture and susceptibility testing. In these scenarios, it is crucial that practitioners are aware of the most common pathogens identified in pneumonia, and the spectrum of activity of the drugs that they prescribe. *Streptococcus equi* subsp. *zooepidemicus* is the most common bacterial species isolated from bacterial pneumonia in adult horses- and is generally susceptible to cephalosporins and penicillin with varying susceptibility to trimethoprim sulfonamide combinations (1, 4, 14, 16, 26, 35–37). Correspondingly, beta lactams (41.8%, *n* = 102/244), ceftiofur sodium (20.9%, *n* = 68/244), and ceftiofur crystalline-free acid (16.4%, *n* = 40/244) were commonly reported as being prescribed as a first line antimicrobial “always” or “most of the time” in this study (Figure 8). Interestingly, aminoglycosides were the class of drugs with the largest percentage of respondents (58.2%, *n* = 142/244) that reported using them as a first line drug “always” or “most of the time” despite frequent resistance of *Streptococcus equi* subsp. *zooepidemicus* to this class (1, 38). It is plausible that the high frequency with which aminoglycosides were prescribed was due to their synergism with beta lactams (cephalosporins and penicillins) and the tendency for practitioners to administer these classes concurrently. In addition to infections with *Streptococcus equi* subsp. *zooepidemicus*, polymicrobial infections with Gram positive, Gram negative and anaerobic bacteria occur in close to half of cases of equine pneumonia, and aminoglycosides have good activity against Gram negative bacteria which supports their use (1, 2, 20). Similar to a previous report on antimicrobial prescribing trends in a university ambulatory practice where sulfonamides and aminoglycosides were the most commonly prescribed class of antimicrobials, respondents in ambulatory practice were significantly more likely to prescribe potentiated sulfonamides as a first line antimicrobial agent when compared to private referral hospitals (*p* = 0.001), though not more than respondents in academic hospitals (8). There was no significant difference between specialists and general practitioners in the frequency with which they prescribed potentiated sulfonamides.

Anaerobic infections occur in 14 to 56% percent of cases and have been associated with non-survival in some studies, yet 42.6% (*n* = 104/244) of respondents reported that they “never” or “sometimes” prescribe metronidazole as a first line antimicrobial, and only 27.8% (*n* = 68/244) of respondents reported that they prescribe it “most of the time” or “always” (1, 2, 5, 20, 39). While penicillin generally has good activity against anaerobes, *Bacteroides* spp., one of the most common anaerobic isolates in pneumonia, are often resistant yet are susceptible to metronidazole (1, 4, 40). Hallmarks of anaerobic infection include putrid odors associated with nasal discharge and the presence of gas within pleural effusions (4, 20, 40, 41). Practitioners that identify these signs should consider adding metronidazole to empiric therapy to potentially improve outcome.

The classes of antimicrobials prescribed varied substantially by country of the respondent. Practitioners in Europe were significantly more likely to prescribe beta lactams aside from cephalosporins than practitioners in the United States. The Committee for Medicinal Products for Veterinary Use (CVMP) in Europe recommends that 3rd and 4th generation cephalosporins and fluoroquinolones be reserved for veterinary patients that have not had the desired response to another antimicrobial agent or are expected not to respond (42). Accordingly, in a survey of antimicrobial use by 264 equine veterinarians from Europe and the UK respiratory disease (including pleuropneumonia and pleuritis) was only considered justification for use of fluoroquinolones and 3rd and 4th generation cephalosporins in a small percentage of respondents (5.1 and 13%, respectively) (43). Despite the classification of 3^rd^ generation cephalosporins as critically important antimicrobials for human medicine by the World Health Organization (WHO), no such guidelines exist for equine veterinarians in the United States (44). Ceftiofur sodium and ceftiofur CFA were commonly prescribed as a first line antimicrobial by practitioners in the United States though interestingly, there were no significant differences in the frequency of respondent’s use of fluoroquinolones between Europe and the United States or other locations.

The ideal duration of antimicrobial therapy in equine pneumonia has not been determined, though a minimum of 10 days of therapy has been suggested (4). However, 50.4% (*n* = 123/244) of respondents in this study reported they typically treat for >2–4 weeks. In human medicine, current guidelines from the American Thoracic Society and Infectious Diseases Society of America recommend 5 days of antimicrobial therapy for most patients with community-acquired pneumonia (7). In 439 children treated for community-acquired pneumonia, there was no significant difference in treatment failure rates between children that received a 5–7 day course of antimicrobials and those that received an 8–14 day course—indicating that a longer course of therapy does not improve outcome (9). Four to 6 weeks of antimicrobial therapy has previously been recommended when treating bacterial pneumonia in dogs and cats, but the Antimicrobial Guidelines Working Group of the International Society for Companion Animal Infectious Diseases cited that there is limited evidence to support this recommendation and in dogs with uncomplicated bacterial pneumonia there was no significant difference in outcome between animals that received a 10-day vs. a 21 day course of antimicrobial therapy (6, 10). Instead, they recommend treating dogs and cats for 10–14 days and reevaluating the clinical, hematologic, and radiographic findings to determine if a longer course of therapy is warranted (6).

Based on evidence in other species and recommendations of the WHO, reducing the duration of antimicrobial therapy for treatment of bacterial pneumonia is likely justified in many cases of pneumonia in horses to reduce antimicrobial resistance and minimize adverse effects of prolonged antimicrobial therapy (34). When survey participants were asked which criteria they used to determine when to discontinue antimicrobial therapy, normalization of WBC count or neutrophil count and resolution of fever were ranked most highly. However, in adult horses with confirmed pneumonia, 44% (*n* = 45/104) and 34% (*n* = 32/94) had WBC and neutrophil counts, respectively, within reference range at the time of presentation and fever was only present in 26% (*n* = 28/110) of animals, suggesting that these parameters are not ideal metrics by which to monitor response to antimicrobial therapy (1). Resolution of ultrasonographic changes of the lung was ranked third, likely due to the accessibility of portable US units to most equine practitioners, making thoracic ultrasonography an attractive tool for the monitoring of pneumonia. Despite widespread use of ultrasonography for monitoring pneumonia, the rate at which ultrasonographic changes of the lung improve in horses with antimicrobial therapy is unknown. In a small study of dogs with aspiration pneumonia, B-lines (comet tails) on ultrasonography and radiographic changes in the lung persisted 1 month after initiation of therapy, despite clinical improvement. Additionally, normal horses that have undergone anesthesia and horses with equine asthma syndrome can have comet tails visible on thoracic ultrasonography in the absence of pneumonia (45–48). Shred signs (defined as irregularity of the normal pulmonary-pleural interface within consolidated lung), however, resolved in the majority of dogs by 1 month after diagnosis (49, 50). In human medicine, ultrasonography has been suggested as a monitoring tool to attempt to reduce the duration of antimicrobial therapy, though there are not sufficient data to support its use as the only monitoring tool (51). While more research is necessary, it is probable that administering antimicrobials until complete resolution of ultrasonographic changes of the lung unnecessarily extends the duration of treatment and may not be indicated.

This study had multiple limitations. All information was self-reported by veterinarians, thus there was potential for bias or false reporting of information. No specific definition of pneumonia was given to respondents, thus some of the cases used in respondents answers may not have been confirmed as pneumonia. Additionally, the survey was developed by a specialist and as such may have asked questions in a way that may have implied certain answers were “correct.” One question (Question 14, Supplementary material S1) was changed to include one additional answer choice shortly after the survey went live in response to feedback from respondents, though this was unlikely to have biased results as only 15.6% (*n* = 36/244) of respondents selected the additional answer choice. While the survey was distributed on multiple social media groups and via several professional organizations, the demographics of the respondents may have been geographically biased by the network of the author. The majority of the respondents practiced within the USA; thus findings should be applied to groups of practitioners in other countries with caution. Lastly, the format of the survey primarily only allowed for selection of the responses provided and offered very few opportunities to supply additional information, such that some respondents may have felt compelled to answer a question a certain way when the answer may not be entirely reflective of their practice.

This study provides a unique description of the methods and therapies that equine veterinarians use to diagnose and treat bacterial pneumonia in adult horses. It identified that there are significant differences in the diagnostic tools and therapies used in the field vs. in a hospital setting. Survey responses provided insight into ways that equine veterinarians can improve antimicrobial stewardship by showing which classes of antimicrobials are used most, and how the duration of therapy frequently exceeds that which is recommended for pneumonia in other species. Furthermore, these data provide a foundation upon which future research in the field of bacterial pneumonia in horses can be based.

## Data Availability

The raw data supporting the conclusions of this article will be made available by the authors, without undue reservation.
