# Supplementary material for: Survey of the approach to the diagnosis and management of bacterial pneumonia in adult horses by equine veterinarians
Source: Front Vet Sci. 2024 Dec 23;11:1484970. doi: 10.3389/fvets.2024.1484970 (PMC11700971; doi:10.3389/fvets.2024.1484970)
Supplement: Supplementary file 1 [file Table_1.DOCX]

Survey of diagnostics and therapy for pneumonia in adult horses

Survey Flow

Practice Information

Q1 How many years have you been a practicing equine veterinarian?

- 0-5 years (1)
- 6-10 years (2)
- 11-15 years (3)
- 16-20 years (4)
- Greater than 20 years (5)

Q2 Approximately what percentage of your practice is equine?

- 0-25% (1)
- 26-50% (2)
- 51-75% (3)
- 76-100% (4)

Q3 What type of practice do you work in?

- All ambulatory (1)
- Ambulatory with hospital facility (2)
- Private practice- hospital/ referral (3)
- Academia-hospital/ referral (4)
- Other, please specify. (5) __________________________________________________

Q4 Which of the following options best describes you?

- General practitioner- no internship (1)
- General practitioner- one year internship after graduation (2)
- Specialist- ACVIM large animal internal medicine diplomate (3)
- Specialist- ACVS large animal surgery diplomate (4)
- Specialist- other (5)
- Other-please specify (6) __________________________________________________

Q5 Where is your primary practice location?

- United States- Northeast (1)
- United States- Midwest (2)
- United States- South (3)
- United States- West (4)
- Europe (5)
- Asia (6)
- South or Central America (7)
- Africa (8)
- Canada (9)
- Australia (10)
- Other- please specify (11) __________________________________________________

Q7 What type of horse is seen most commonly in your practice?

- Pleasure horse (eg trail riding, companion animal) (1)
- Racehorses (2)
- Western performance horses (eg Quarter Horse and Paint events) (3)
- English performance horses (eg dressage, hunter jumper, 3-day eventing) (4)
- Breeding animals (5)
- Gaited performance horses (eg Saddlebred, Tennessee Walking Horses, etc) (6)
- Working animals (eg ranch horses, cart horses, etc) (7)
- Other-please specify (8) __________________________________________________

Clinical Findings

Q9 Please rate the following items from low to high based on how big of a risk factor they are for pneumonia in adult horses.

|  | No risk (1) | Low risk (2) | Moderate risk (3) | High risk (4) |
| --- | --- | --- | --- | --- |
| Esophageal obstruction (choke) (1) |  |  |  |  |
| Trailer transport 0-3 hours (without stops) (2) |  |  |  |  |
| Trailer transport 4-6 hours (without stops) (3) |  |  |  |  |
| Trailer transport >6 hours (without stops) (4) |  |  |  |  |
| Non-viral upper respiratory disease (eg strangles, guttural pouch disease, laryngeal paralysis) (5) |  |  |  |  |
| General anesthesia (inhalant) (6) |  |  |  |  |
| General anesthesia (injectable) (7) |  |  |  |  |
| Transport via air (8) |  |  |  |  |
| Viral upper respiratory infection (eg Equine Herpes Virus 1, Equine Influenza Virus) (9) |  |  |  |  |
| Equine asthma syndrome (10) |  |  |  |  |

Q10 How often do you see the following clinical signs in adult horses with pneumonia?

|  | Never (1) | Sometimes (2) | About half the time (3) | Most of the time (4) | Always (5) |
| --- | --- | --- | --- | --- | --- |
| Nasal discharge (1) |  |  |  |  |  |
| Cough (2) |  |  |  |  |  |
| Tachypnea/ dyspnea/increased respiratory effort (3) |  |  |  |  |  |
| Fever (rectal temperature >101.5 F, 38.6 C) (4) |  |  |  |  |  |
| Weight loss (5) |  |  |  |  |  |
| Colic (eg pawing, rolling, biting at sides) (6) |  |  |  |  |  |
| Ventral edema (7) |  |  |  |  |  |
| Tachycardia (heart rate >48 beats per minute) (8) |  |  |  |  |  |
| Lethargy (9) |  |  |  |  |  |
| Anorexia (10) |  |  |  |  |  |

Q12 What is the most common presenting complaint from owners of adult horses in which you have diagnosed pneumonia?

- Cough (1)
- Weight loss (2)
- Exercise intolerance (3)
- Anorexia (4)
- Nasal discharge (5)
- Tachypnea/ dyspnea/ increased respiratory effort (6)
- Colic (7)
- Weight loss (8)
- Lethargy (9)
- Edema (10)
- Other- please specify (11) __________________________________________________

Q13 How often do you hear the following sounds on lung auscultation in adult horses with pneumonia?

|  | Never (1) | Rarely (2) | About half of the time (3) | Most of the time (4) | Always (5) |
| --- | --- | --- | --- | --- | --- |
| Crackles (1) |  |  |  |  |  |
| Wheezes (2) |  |  |  |  |  |
| Reduced to absent lung sounds (3) |  |  |  |  |  |
| Normal bronchovesicular sounds (4) |  |  |  |  |  |
| Pleural rubs (5) |  |  |  |  |  |

Diagnosis of Pneumonia in Adult Horses

Q24 What criteria or diagnostic modalities do you use to diagnose pneumonia in adult horses? Please select all that apply.

- Physical exam findings (eg cough, fever, nasal discharge) (1)
- Thoracic ultrasound (2)
- Thoracic radiography (3)
- Bacterial growth on transtracheal wash (4)
- Suppurative inflammation on transtracheal wash cytology (5)
- Endoscopy (6)
- Inflammatory leukogram or hyperfbrinogenemia on CBC (7)
- Increased SAA (8)
- Other (9) __________________________________________________

Q15 How likely are you to do the following diagnostics when you suspect pneumonia?

|  | Extremely unlikely (1) | Somewhat unlikely (2) | Neither likely nor unlikely (3) | Somewhat likely (4) | Extremely likely (5) |
| --- | --- | --- | --- | --- | --- |
| Complete blood count (1) |  |  |  |  |  |
| Chemistry panel (2) |  |  |  |  |  |
| Lactate (3) |  |  |  |  |  |
| Serum amyloid A (4) |  |  |  |  |  |
| Venous blood gas (5) |  |  |  |  |  |
| Arterial blood gas (6) |  |  |  |  |  |
| Rebreathing exam (7) |  |  |  |  |  |
| Thoracic percussion (8) |  |  |  |  |  |
| Thoracic ultrasound (9) |  |  |  |  |  |
| Endoscopy (10) |  |  |  |  |  |
| Thoracic radiography (11) |  |  |  |  |  |
| Bronchoalveolar lavage (12) |  |  |  |  |  |
| Thoracocentesis (13) |  |  |  |  |  |
| Transtracheal wash with cytology and culture (14) |  |  |  |  |  |
| Transtracheal wash with cytology only (15) |  |  |  |  |  |
| Transtracheal wash with culture only (16) |  |  |  |  |  |
| Nasopharygneal swab culture (17) |  |  |  |  |  |
| Nasopharyngeal swab PCR for respiratory viruses (18) |  |  |  |  |  |

| Page Break |  |
| --- | --- |

Q25 How frequently do you perform transtracheal washes when you suspect pneumonia?

- Never (1)
- Sometimes (2)
- About half the time (3)
- Most of the time (4)
- Always (5)

Q16 If you perform transtracheal washes, which method do you use most frequently?

- Endoscopic transtracheal wash (1)
- Percutaneous transtracheal wash (2)
- I don’t perform transtracheal washes (3)

Q26 How often are your final antimicrobial choices for pneumonia based on culture and susceptibility results?

- Never (1)
- Sometimes (2)
- About half the time (3)
- Most of the time (4)
- Always (5)

Therapy for Pneumonia

Q17 How frequently do you use the following antimicrobial classes or drugs as a first-line therapy for confirmed or suspected pneumonia (prior to culture results if performed) in an adult horse?

|  | Never (1) | Sometimes (2) | About half the time (3) | Most of the time (4) | Always (5) |
| --- | --- | --- | --- | --- | --- |
| Sulfonamide/ trimethoprim combinations (1) |  |  |  |  |  |
| Fluroquinolones (2) |  |  |  |  |  |
| Ceftiofur sodium (3) |  |  |  |  |  |
| Ceftiofur crystalline-free acid (4) |  |  |  |  |  |
| Cephalosporin (excluding ceftiofur) (5) |  |  |  |  |  |
| Beta lactams (6) |  |  |  |  |  |
| Aminoglycosides (7) |  |  |  |  |  |
| Metronidazole (8) |  |  |  |  |  |
| Macrolides (9) |  |  |  |  |  |
| Chloramphenicol (10) |  |  |  |  |  |
| Tetracyclines (11) |  |  |  |  |  |
| Carbapenems (12) |  |  |  |  |  |

Q25 Which route of antimicrobial administration are you most likely to use for initial treatment of pneumonia?

- Intravenous (1)
- Oral (2)
- Intramuscular (3)
- Inhaled/nebulized (4)
- Rectal (5)

Q18 How long do you typically treat adult horses with bacterial pneumonia with antimicrobials?

- < 1 week (1)
- 1-2 weeks (2)
- >2 weeks-4 weeks (3)
- >4-8 weeks (4)
- > 8 weeks (5)

Q21 Which variable do you use most frequently to guide duration of antimicrobial therapy? Please rank these items from 1 (most likely) to lowest (9-least likely).

______ Normalization of white cell count or neutrophil count (1)

______ Normalization of fibrinogen (2)

______ Normalization of SAA (3)

______ Resolution of ultrasonographic changes in the lung (4)

______ Resolution of radiographic changes in the lung (5)

______ Resolution of fever (6)

______ Resolution of cough (7)

______ No bacterial growth on repeated transtracheal wash (8)

______ Other-please specify (9)

Q18 How frequently do you use the following adjunct therapies in addition to systemic antimicrobials?

|  | Never (1) | Sometimes (2) | About half the time (3) | Most of the time (4) | Always (5) |
| --- | --- | --- | --- | --- | --- |
| Intravenous fluid therapy (1) |  |  |  |  |  |
| NSAIDs (2) |  |  |  |  |  |
| Polymixin B (3) |  |  |  |  |  |
| Digital cryotherapy (4) |  |  |  |  |  |
| Intranasal oxygen (5) |  |  |  |  |  |
| Systemic corticosteroids (6) |  |  |  |  |  |
| Pentoxifylline (7) |  |  |  |  |  |
| Anticoagulants (8) |  |  |  |  |  |
| Plasma transfusion (9) |  |  |  |  |  |
| Inhaled corticosteroids (10) |  |  |  |  |  |
| Inhaled antimicrobials (11) |  |  |  |  |  |
| Systemic bronchodilators (12) |  |  |  |  |  |
| Inhaled bronchodilators (13) |  |  |  |  |  |
| Non-NSAID analgesics (eg opioids, lidocaine, etc) (14) |  |  |  |  |  |

Q19 In cases of pleuropneumonia (defined as accumulation of fluid in the pleural space), please rate how often you use the following therapies.

|  | Never (1) | Rarely (2) | About half of the time (3) | Most of the time (4) | Always (5) |
| --- | --- | --- | --- | --- | --- |
| Thoracic lavage with cystalloids (1) |  |  |  |  |  |
| Intrathoracic antimicrobials (2) |  |  |  |  |  |
| Intrathoracic tissue plasminogen activator (3) |  |  |  |  |  |
| Intrathoracic acetylcysteine (4) |  |  |  |  |  |
| Intrathoracic platelet lysate (5) |  |  |  |  |  |
| Intermittent thoracocentesis and drainage (6) |  |  |  |  |  |
| Indwelling thoracic drain (chest tube) (7) |  |  |  |  |  |
| Thoracotomy (8) |  |  |  |  |  |

Q26 How comfortable are you with performing the following procedures?

|  | Extremely uncomfortable (1) | Somewhat uncomfortable (2) | Neither comfortable nor uncomfortable (3) | Somewhat comfortable (4) | Extremely comfortable (5) |
| --- | --- | --- | --- | --- | --- |
| Thoracic ultrasound (1) |  |  |  |  |  |
| Transtracheal wash (2) |  |  |  |  |  |
| Thoracocentesis (3) |  |  |  |  |  |
| Bronchoalveolar lavage (4) |  |  |  |  |  |
